# Effects of Chronic Low-Salinity Stress on Growth, Survival, Antioxidant Capacity, and Gene Expression in *Mizuhopecten yessoensis*

**DOI:** 10.3390/biology14070759

**Published:** 2025-06-25

**Authors:** Haoran Xiao, Xin Jin, Zitong Wang, Qi Ye, Weiyan Li, Lingshu Han, Jun Ding

**Affiliations:** 1 Liaoning Provincial Key Laboratory of Northern Aquatic Germplasm Resources and Genetics and Breeding, Dalian Ocean University, Dalian 116023, China; 2 Key Laboratory of Mariculture & Stock Enhancement in North China’s Sea, Ministry of Agriculture and Rural Affairs, Dalian Ocean University, Dalian 116023, China; 3 Dalian Jinshiwan Laboratory, Dalian 116034, China

**Keywords:** *Mizuhopecten yessoensis*, low salinity, growth performance, antioxidant defense, transcriptomics

## Abstract

Extreme weather events such as heavy rainfall significantly reduce surface salinity in coastal waters, presenting considerable challenges to the aquaculture of Japanese scallops (*Mizuhopecten yessoensis*) in shallow cage systems. This study investigated the effects of chronic low-salinity stress on the growth performance, antioxidant capacity, and gene expression profile of *M. yessoensis* using a 60-day salinity gradient experiment. S33 represents the control treatment with normal seawater salinity (33‰), while S30, S28, and S26 represent experimental groups with progressively lower salinities of 30‰, 28‰, and 26‰, respectively. A decline in salinity was accompanied by an increase in oxygen consumption. The S26 group exhibited a higher ammonia excretion rate (2.73 μg/g·h) than other groups, indicating intensified nitrogen metabolism. Growth was inhibited under low-salinity conditions. The S33 group exhibited greater weight gain (16.7%) and shell growth (8.4%) compared to the S26 group (11.6% and 6%), which also showed a substantially higher mortality rate (46%) compared to the control (13%). At 28‰, antioxidant enzyme activities (T-AOC, SOD, CAT, POD) were elevated, indicating a moderate level of stress. However, at the lowest salinity (26‰), these indicators decreased, reflecting the exhaustion of the antioxidant systems and indicating that the mollusks’ adaptive capacity had been exceeded, leading to a state of stress fatigue. NAD-MDH activity was elevated in the S26 group, reflecting enhanced aerobic metabolism under stress. Transcriptome analysis revealed 564 differentially expressed genes (DEGs) between the S33 and S26 groups. Functional enrichment analysis indicated that these DEGs were mainly associated with immune and stress response pathways, including NF-κB, TNF, apoptosis, and Toll/Imd signaling. These genes are involved in key metabolic processes, such as alanine, aspartate, and glutamate metabolism. Genes such as *GADD45*, *ATF4*, *TRAF3*, and *XBP1* were upregulated, contributing to stress repair and antioxidant responses. Conversely, the expressions of *CASP3*, *IKBKA*, *BIRC2/3*, and *LBP* were downregulated, potentially mitigating apoptosis and inflammatory responses. These findings suggest that *M. yessoensis* adapts to chronic low-salinity stress through the activation of antioxidant systems, modulation of immune responses, and suppression of excessive apoptosis. This study provides new insights into the molecular mechanisms underlying salinity adaptation in bivalves and offers valuable references for scallop aquaculture and selective breeding programs.

## Simple Summary

Rain and other extreme weather can lower salt levels in coastal waters, making it harder to farm Japanese scallops. This study tested how scallops respond to low-salt water over time. When salt levels dropped too much, scallops grew poorly, used more energy, and many died. Some were able to adjust at moderate salt levels, but at very low levels, their defenses stopped working. The study also looked at changes in gene activity to understand how scallops respond to stress. These results can help improve scallop farming by showing which salt levels are safe and which should be avoided.

## 1. Introduction

*Mizuhopecten yessoensis* is a large cold-water bivalve widely distributed along the coastal waters of Northeast Asia. It is considered a major species in regional aquaculture. In northern coastal China, it is regarded as one of the most important cultivated bivalves [1]. This species also plays a significant ecological role. As a filter-feeding mollusk, it helps purify water by consuming phytoplankton. Its carbon-rich shell and tissues contribute to long-term CO_2_ sequestration, thus supporting marine carbon sink functions [2,3]. Previous studies have shown that filter-feeding bivalves have high carbon content. Consequently, the expansion of coastal bivalve aquaculture may contribute to marine carbon fixation [4]. Therefore, *M. yessoensis* holds not only economic value for fisheries but also ecological significance as a key species in carbon sequestration and ecosystem functioning [5].

In recent years, increasing fluctuations in surface salinity in nearshore waters have become a prominent feature of coastal aquaculture environments [6]. These fluctuations are primarily attributed to the increasing frequency of extreme weather events—such as heavy rainfall and prolonged drought—driven by global climate change. Such events may cause abrupt and localized changes in salinity, imposing physiological stress on marine organisms and, under severe conditions, resulting in mass mortality [7,8]. For *M. yessoensis*, which inhabits surface waters in suspended raft cages, sudden drops in salinity following rainstorms may trigger acute low-salinity stress [9,10]. This species is highly sensitive to salinity variation. When salinity levels temporarily fall below its tolerance range (25‰), it may experience stress responses and physiological disorders that seriously threaten its survival [11]. Consequently, the growing prevalence of low-salinity conditions in coastal waters presents a critical challenge for the sustainable cultivation of *M. yessoensis*. This highlights the importance of understanding its physiological and molecular responses to salinity stress.

Salinity fluctuations exert profound effects on the physiological metabolism and immune–oxidative functions of bivalves. When salinity deviates from optimal levels, bivalves often adjust their metabolic activity to maintain osmotic balance. These adjustments are typically reflected in changes in energy metabolism indicators, such as oxygen consumption and ammonia excretion rates. Disruptions in these processes may lead to energy imbalance, growth impairment, or even mortality [12]. Moreover, low-salinity stress is often accompanied by oxidative damage and immune activation [13]. Sudden changes in salinity are known to disrupt the physiological balance of bivalves, particularly through the induction of oxidative stress and immune dysfunction. Several studies have shown that acute low-salinity exposure significantly alters key antioxidant and immune indicators. For instance, under salinity shock, oysters exhibit changes in the activities of antioxidant enzymes such as superoxide dismutase (SOD), catalase (CAT), and peroxidase (POD) alongside fluctuations in immune-related enzymes like alkaline phosphatase (AKP) and lysozyme (LSZ) [14]. Additionally, levels of malondialdehyde (MDA), a marker of lipid peroxidation and oxidative damage, can increase dramatically—up to 100-fold in extreme cases [15]. While these physiological changes clearly reflect stress responses, interpreting them solely based on linear cause–effect models may oversimplify the underlying mechanisms. In fact, increasing evidence in redox biology suggests that the relationship between oxidative stress and cellular response is often non-linear and biphasic. This phenomenon, commonly referred to as hormesis or a bell-shaped response, posits that low to moderate levels of reactive oxygen species (ROS) can activate beneficial adaptive mechanisms—such as the upregulation of antioxidant defenses and stress-responsive genes—whereas excessive ROS production overwhelms the system, leading to cellular damage, inflammation, and apoptosis [16,17,18].

To better understand how bivalves adapt to salinity stress, recent studies have explored their molecular responses to low-salinity conditions. Evidence suggests that bivalves or mollusks activate various stress-related signaling pathways and gene expression responses under low-salinity exposure [19,20]. Transcriptomic analyses indicate that low-salinity stress leads to the reprogramming of key biological pathways. These include NF-κB and TNF signaling pathways, which are involved in innate immunity and inflammation, as well as apoptosis and energy metabolism pathways, such as the tricarboxylic acid (TCA) cycle and amino acid metabolism [21]. For example, under low-salinity conditions, bivalves may improve survival by regulating apoptosis-related gene expression. Anti-apoptotic genes, such as those encoding BIR domain-containing proteins (*BIRC2/3*), are often upregulated, whereas pro-apoptotic genes like caspase-3 (*CASP3*) may be downregulated. This expression profile suggests that marine organisms may adapt to salinity stress by limiting excessive apoptosis [21].

However, most existing studies have concentrated on the physiological, biochemical, or molecular responses to acute or short-term salinity stress, while much less is known about how bivalves respond to chronic low-salinity conditions. In particular, comprehensive transcriptomic analyses examining long-term gene expression changes under extended low-salinity exposure remain scarce. This knowledge gap limits our understanding of the mechanisms underlying salinity tolerance in bivalves. It also constrains the development of effective aquaculture management strategies and targeted selective breeding programs.

In light of these challenges, the present study investigated the responses of *M. yessoensis* to chronic low-salinity stress using a 60-day exposure experiment. High-throughput RNA sequencing (RNA-seq) was employed to systematically profile transcriptomic changes in scallops subjected to prolonged low-salinity exposure. The analysis focused on the regulation of antioxidant enzyme activity, modulation of apoptosis-related signaling pathways, and reprogramming of energy metabolism. By examining adaptive responses in oxidative defense, apoptotic regulation, and metabolic adjustment, this study aims to elucidate the physiological and molecular mechanisms by which *M. yessoensis* adapts to chronic low-salinity stress. The findings will provide a theoretical foundation for improving environmental management practices and advancing the development of salinity-tolerant scallop strains.

## 2. Materials and Methods

### 2.1. Ethics Statement

*M. yessoensis* individuals used in this study were all artificially bred from the same cohort in Xiaoyaowan, Jinzhou Development Zone, Dalian, China. All experimental procedures were conducted in strict accordance with the relevant national guidelines of China and the institutional regulations of Dalian Ocean University.

### 2.2. Experimental Animals and Stress Design

The salinity stress experiment was conducted at the Key Laboratory of Mariculture, Ministry of Agriculture, Dalian Ocean University. All experimental *M. yessoensis* individuals were 1.5 years old. Prior to the experiment, surface debris was removed from the *M. yessoensis*, and sand-filtered seawater was prepared for the stress treatments. The scallops were maintained in a recirculating aquaculture system and acclimated by feeding *Chlorella* for one week. A total of 180 healthy individuals were randomly assigned to 12 square tanks (100 L each), with 15 scallops per tank. Prior to the experiment, the shell length, shell width, shell height, and wet weight were measured for each individual. *Chlorella* was administered twice daily at 7:00 a.m. and 4:30 p.m. Seawater was renewed every three days via siphoning, and feces were removed on a daily basis. The rearing conditions were maintained under low-light intensity, with the natural seawater temperature gradually declining from 18 °C to 13 °C. The pH was maintained between 7.6 and 8.3; dissolved oxygen levels were kept above 6 mg/L; and ammonia nitrogen and nitrite concentrations were maintained below 0.05 mg/L.

Chronic low-salinity stress was simulated through the proportional addition of distilled water, reducing salinity by 2‰ every three days to avoid abrupt drops at the beginning of the experiment. Four salinity levels were established: 33‰, 30‰, 28‰, and 26‰, designated as S33, S30, S28, and S26, respectively. Since the salinity of normal seawater is around 33‰, salinity of 33‰ was set as the control group. Each salinity treatment group included three replicates. The experiment lasted for 60 days, during which salinity was adjusted using a mixture of seawater and distilled water and monitored with a salinometer.

### 2.3. Sample Collection

At the beginning and end of the experiment, 100 mL of seawater was collected from each tank to measure the dissolved oxygen consumption and ammonia excretion rates. Upon completion of the experiment, all *Mizuhopecten yessoensis* individuals in each tank were measured for shell length, shell width, shell height, wet weight, soft tissue weight, and shell weight. Subsequently, ten individuals were randomly selected from each tank for tissue sampling. The soft tissue, adductor muscle, and visceral mass were dissected, immediately flash-frozen in liquid nitrogen, and transferred into 1.5 mL RNase-free microcentrifuge tubes (Axygen, Union City, CA, USA). Samples were stored at −80 °C for subsequent enzyme activity assays, gene expression analysis, and transcriptomic sequencing.

### 2.4. Growth and Survival Assessment

During the experiment, we considered the scallops dead if their shells remained open and could not close again. These individuals were included in the mortality count. At the end of the experiment, we measured the shell length, shell width, and shell height of each scallop using a vernier caliper. The wet weight, soft body weight, and shell weight of each scallop were measured using an electronic balance.

### 2.5. Measurement of Oxygen Consumption and Ammonia Excretion Rates

Dissolved oxygen (DO) levels at the start and end of the experiment were recorded using a HACH water quality analyzer. Ammonium nitrogen (NH_4_^+^-N) concentrations were determined using the Nessler’s reagent method, appropriate for seawater.

The oxygen consumption rate (RO) and ammonia excretion rate (RN) per unit dry mass were calculated using the following formulas:

RO = [(Initial DO − Final DO) × Volume]/(Dry Mass × Time)

RN = [(Final NH_4_^+^-N − Initial NH_4_^+^-N) × Volume]/(Dry Mass × Time)

where DO and NH_4_^+^-N concentrations (mg/L or μmol/L) were measured at the start and end of the experiment, volume represents the volume of water (L), dry mass is the soft tissue mass (g), and time refers to the total experimental duration (h).

### 2.6. Enzyme Activity Assays

Visceral mass tissues collected at the end of the experiment were homogenized for antioxidant and immune enzyme assays. The parameters included catalase (CAT), peroxidase (POD), superoxide dismutase (SOD), total antioxidant capacity (T-AOC), malondialdehyde (MDA) content, NAD-dependent malate dehydrogenase (NAD-MDH) activity, and total protein content. All assays were conducted using commercial kits (Nanjing Jiancheng Bioengineering Institute) according to the manufacturer’s protocols.

### 2.7. Sample Collection and RNA Extraction

RNA Isolation and cDNA Synthesis: Total RNA was extracted from the body wall of *M. yessoensis* by using TianGen’s RNA Easy Fast kit (DP451) and FastKing gDNA Dispelling RT SuperMix (KR118) (TianGen, Beijing, China) [22]. The RNA integrity was confirmed via agarose gel electrophoresis by using 50X TAE Buffer (B548101) and Agarose, Regular (A620014) (Sangon Biotech, Shanghai, China) [23]. cDNA synthesis was performed under the following conditions: incubation at 37 °C for 15 min, denaturation at 85 °C for 5 s, and storage of the cDNA at −20 °C.

Primer Design: Primers for RT-PCR were devised using NCBI’s Primer BLAST (2.15.0), targeting specific sequences within the coding regions identified in the NCBI database. This rigorous approach yielded eight pairs of primers, including the reference gene primer Cytochrome b (Cytb-R), cited directly from the relevant literature [24] (Table 1).

Quantitative PCR Setup and Analysis: The qPCR was performed by using the FastKing One Step RT-PCR Kit (KR123) (TianGen, Beijing, China) [25]. The qPCR employed SYBR Green I chemistry in a 20 μL reaction mixture containing 10 μL 2 × TransStart^®^ Tip Green qPCR Super Mix, 0.8 μL each of forward and reverse primers (20μM), 2 μL of cDNA, and 6.4 μL of RNase-free water. PCR cycling consisted of an initial denaturation at 95 °C for 120 s, followed by 45 cycles of 95 °C for 5 s, and 95 °C for 10 s for annealing/extension, concluding with a melt curve analysis.

Standard Curve and Dilution: Standard curves for the reference and target genes were established using high-expression samples at an initial concentration of 50 ng/μL, followed by a tenfold dilution series across five points, each replicated three times. The optimal reaction cDNA concentration was determined to be 5 ng/μL, achieved by diluting 50 ng/μL cDNA tenfold with DEPC-treated water.

Relative expression levels were calculated using the 2^−ΔΔCT^ method. This involved computing ΔCT as the difference between the CT values of the target and reference genes, normalizing ΔCT to a control group to get ΔΔCT, and determining the fold change as 2^−ΔΔCT^. Results were expressed as the means and standard deviations.

### 2.8. Library Construction and High-Throughput Sequencing

Qualified RNA samples were submitted to Annoroad Gene Technology (Beijing, China) for library preparation and sequencing on an Illumina HiSeq™ 2500 platform (Illumina, San Diego, CA, USA). Poly(A) tails were isolated to enrich mRNA, which was then fragmented and reverse-transcribed to synthesize cDNA. Double-stranded cDNA underwent end repair, A-tailing, adapter ligation, and PCR amplification. Library fragments were approximately 300 bp in length. Sequencing was conducted using a 150 bp paired-end (PE150) strategy, generating over 20 million clean reads per sample (ASM211388v2).

### 2.9. Quality Control and Sequence Assembly

Raw sequence data were assessed using FastQC (v0.11.9). Low-quality reads, adapter sequences, and reads with high N content were removed using Trimmomatic to obtain clean reads. Trinity (v2.15.1) software was used for de novo transcriptome assembly with default parameters. Unigenes were annotated by aligning against the NR, COG/KOG, GO, Swiss-Prot, eggNOG, and KEGG databases. All transcriptome data were deposited in the NCBI database (Accession: PRJNA1263710).

### 2.10. Differential Gene Expression Analysis

Read counts were normalized using DESeq2 (v1.40.2) software. Differentially expressed genes (DEGs) were identified using a false discovery rate (FDR) < 0.05 and |log2FoldChange| > 1. Volcano plots were used for visualization. KEGG pathway enrichment of DEGs was performed using the clusterProfiler (v4.8.2) package, applying Fisher’s exact test and FDR correction.

### 2.11. Data Analysis

Data were analyzed using SPSS 22.0 statistical software. Initially, the normality of data distribution was assessed with the Shapiro–Wilk test, and the homogeneity of variances was evaluated with Levene’s test. Subsequently, one-way analysis of variance (ANOVA) was employed to determine the significance among dietary groups. If significance (*p* < 0.05) was detected, Tukey’s multiple comparisons were used to identify the significant differences between dietary groups. All data were presented as means ± standard errors (SEs).

## 3. Results

### 3.1. Effects of Different Low-Salinity Levels on Shell Length Growth Rate, Wet Weight Gain, and Mortality of M. yessoensis

**Figure 1:**
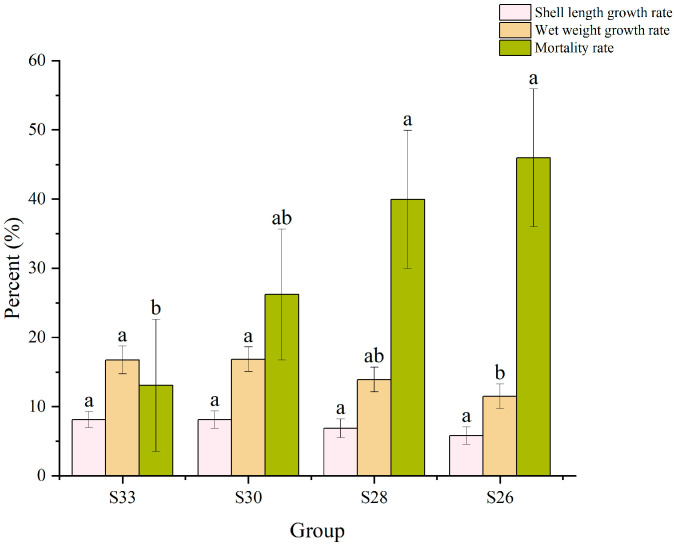
Shell length growth rate, wet weight growth rate, and mortality rate of *Mizuhopecten yessoensis* under low-salt stress. Different letters represent significance (*p* < 0.05), while the same letters do not represent significance (*p* > 0.05).

The effects of different salinity levels on the growth and survival of *M. yessoensis* are shown in Figure 1. No significant differences in shell length were observed among the groups (*p* > 0.05), although a decreasing trend was noted with reduced salinity. The highest shell length growth rate was observed in the S33 group (8.4%) and the lowest in the S26 group (6%). Some groups showed differences in wet weight gain rate (*p* < 0.05); S33 (16.7%) and S30 (16.6%) were higher than S26 (11.6%), and an overall decreasing trend was associated with lower salinity. Mortality increased with declining salinity, with S26 reaching 46%, while the minimum observed value was 13% in the S33 group.

### 3.2. Effects of Different Low-Salinity Levels on Oxygen Consumption and Ammonia Excretion

**Figure 2:**
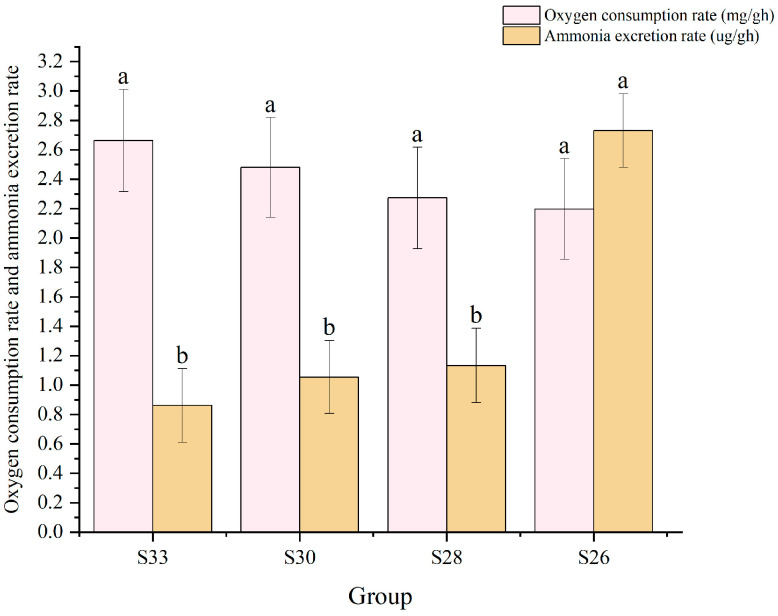
Oxygen consumption rate and ammonia excretion rate of *Mizuhopecten yessoensis* under low-salt stress. Different letters represent significance (*p* < 0.05), while the same letters do not represent significance (*p* > 0.05).

Oxygen consumption and ammonia excretion under different salinity levels are shown in Figure 2. Oxygen consumption showed no significant differences among groups (*p* > 0.05) but tended to increase as salinity decreased. Ammonia excretion showed differences among some groups (*p* < 0.05); in particular, the S26 group had the highest value, reaching 2.73 μg/g·h.

### 3.3. Effects of Different Low-Salinity Levels on Enzyme Activities in M. yessoensis

**Figure 3:**
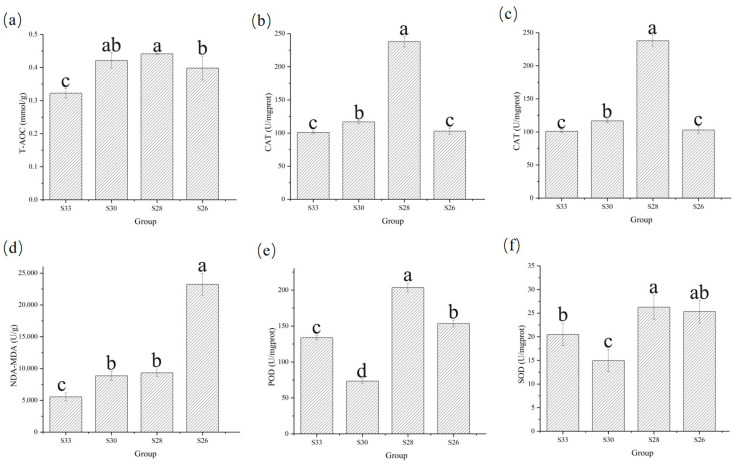
Changes in enzyme activities of *Mizuhopecten yessoensis* under low-salt stress. Different letters indicate statistically significant differences (*p* < 0.05), while the same letters indicate no significant difference (*p* > 0.05). Panel (**a**) shows the activity of T-AOC (Total Antioxidant Capacity), (**b**) shows the activity of CAT (Catalase), (**c**) shows the level of MDA (Malondialdehyde), (**d**) shows the activity of NAD-MDH (NAD-dependent Malate Dehydrogenase), (**e**) shows the activity of POD (Peroxidase), and (**f**) shows the activity of SOD (Superoxide Dismutase).

Under different salinity conditions, significant variations were observed in the activity or content of CAT, MDA, POD, SOD, T-AOC, and NAD-MDH in the visceral mass (Figure 3). CAT activity (Figure 3b) fluctuated with salinity following a “rise–fall–rise–fall” pattern and peaked at 28‰ (238.02 U/mg prot). MDA content (Figure 3c) showed a “rise–fall–rise” trend. No differences were observed between the 28‰ and 30‰ groups (*p* ≥ 0.05). POD activity (Figure 3e) followed a “rise–fall–rise” pattern, with a maximum at 28‰ (203.22 U/mg prot) and a minimum at 30‰. SOD activity (Figure 3f) showed a “fall–rise–fall” pattern, with a peak at 28‰ (27.68 U/mg prot), and the lowest value in the 30‰ group. T-AOC (Figure 3a) exhibited a similar “rise–fall” trend and reached a maximum at 28‰ (0.44 mmol/g), indicating enhanced total antioxidant capacity at this salinity level. NAD-MDH activity (Figure 3d) also followed a fluctuating trend, peaking at 26‰ (22,580.31 U/g), and was higher than in the other groups.

### 3.4. Differential Gene Expression

**Figure 4:**
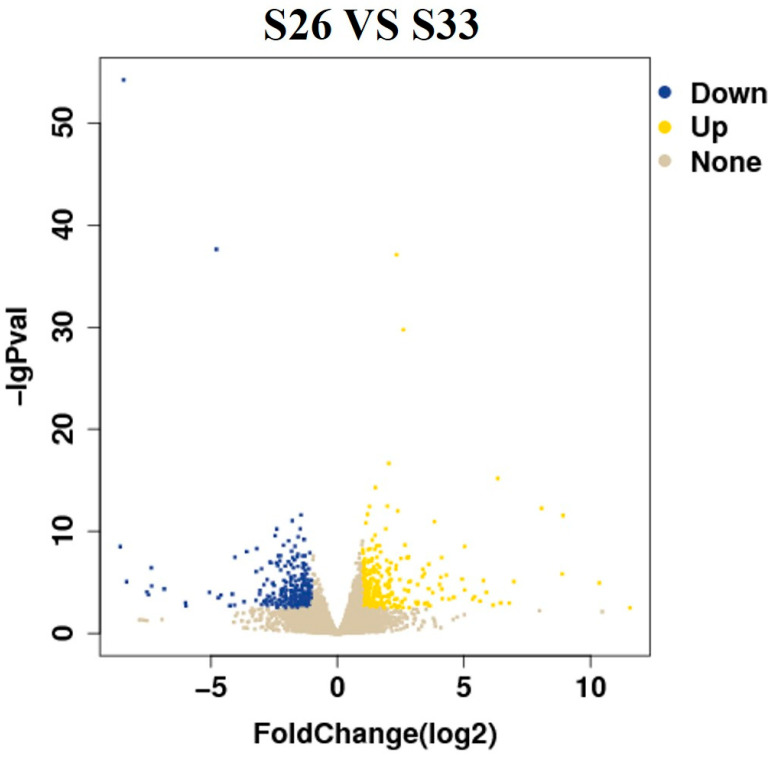
Volcano map of differential genes.

We observed that the S26 group exhibited more severe physiological damage compared to the other experimental groups. Therefore, transcriptomic analysis was conducted between the S26 and S33 groups to investigate the molecular responses under extreme salinity stress. Differentially expressed genes (DEGs) were screened using |log_2_FoldChange| > 1 and *p* < 0.05. As shown in Figure 4, a total of 564 DEGs were identified, including 264 upregulated and 300 downregulated genes.

Representative DEGs and their functions are summarized in Table 2. Key genes included *CASP3*, *IKBKA*, and *BIRC2/3*, involved in NF-κB signaling and apoptosis inhibition; *ABCG1* and *LBP*, associated with lipid transport and LPS recognition; *ATF4* and *GADD45*, linked to stress response and DNA repair; *TRAF3*, participating in TNF receptor signaling; and several metabolism-related genes such as *CAPN1*, *ASNS*, *glnA*, and *gadB*, involved in maintaining osmotic balance and energy metabolism.

### 3.5. KEGG Pathway Enrichment

**Figure 5:**
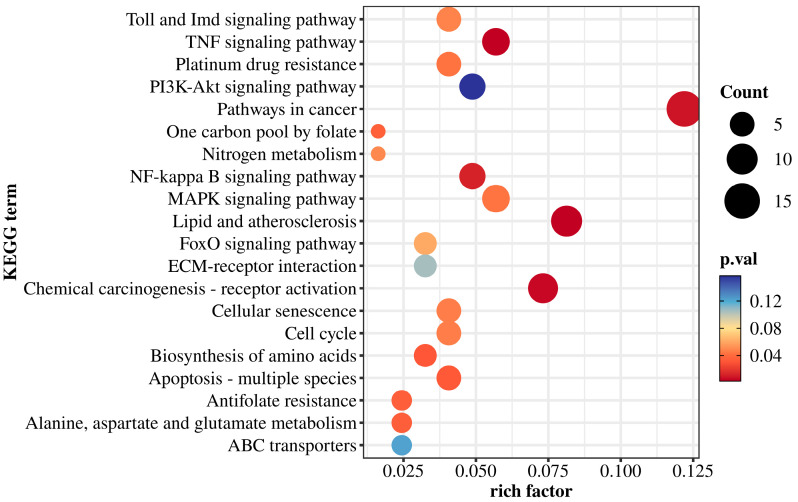
KEGG enrichment bubble plot.

According to the KEGG enrichment results (Figure 5), DEGs were mapped to several signaling and metabolic pathways. Immune and stress-related pathways included the Toll and Imd signaling pathways, TNF signaling pathway, PI3K-Akt pathway, MAPK pathway, NF-κB signaling, and FoxO pathway. These enrichments suggest that low-salinity stress may trigger immune and inflammatory responses in *M. yessoensis*.

Pathways associated with cell fate regulation were also enriched, including cellular senescence, cell cycle, apoptosis (multiple species), and cancer-related pathways. These results imply possible activation of cell cycle arrest and apoptotic mechanisms under low-salinity stress.

Additionally, several metabolic pathways were enriched, such as nitrogen metabolism, one-carbon pool by folate, amino acid biosynthesis, alanine, aspartate and glutamate metabolism, and ABC transporters.

Five pathways were closely related to low-salinity stress response, including Pathways in cancer (map05200), Lipid and atherosclerosis (map05417), Apoptosis (map04210), NF-kappa B signaling (map04064), and TNF signaling (map04668). These pathways contained multiple DEGs with potential biological relevance in oxidative stress, apoptosis, inflammation, and metabolic regulation.

**Figure 6:**
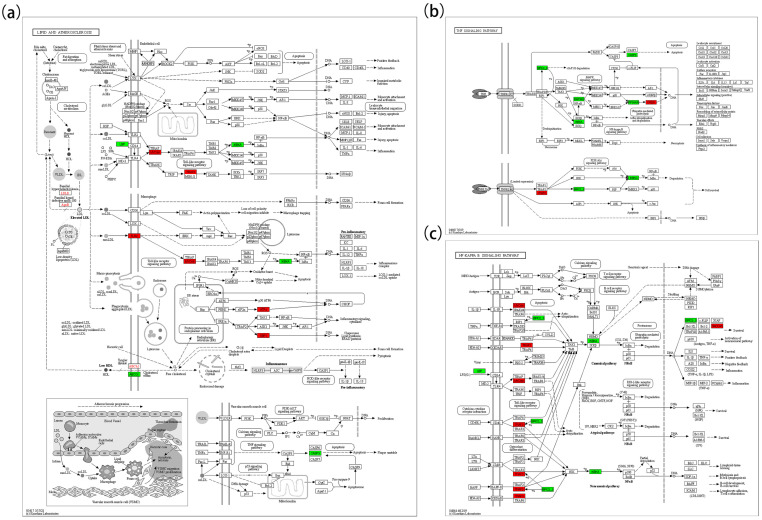
KEGG map 1. (**a**) Represents the Lipid and Atherosclerosis pathway (map05417); (**b**) represents the TNF signaling pathway (map04668); (**c**) represents the NF-κB signaling pathway (map04064).

**Figure 7:**
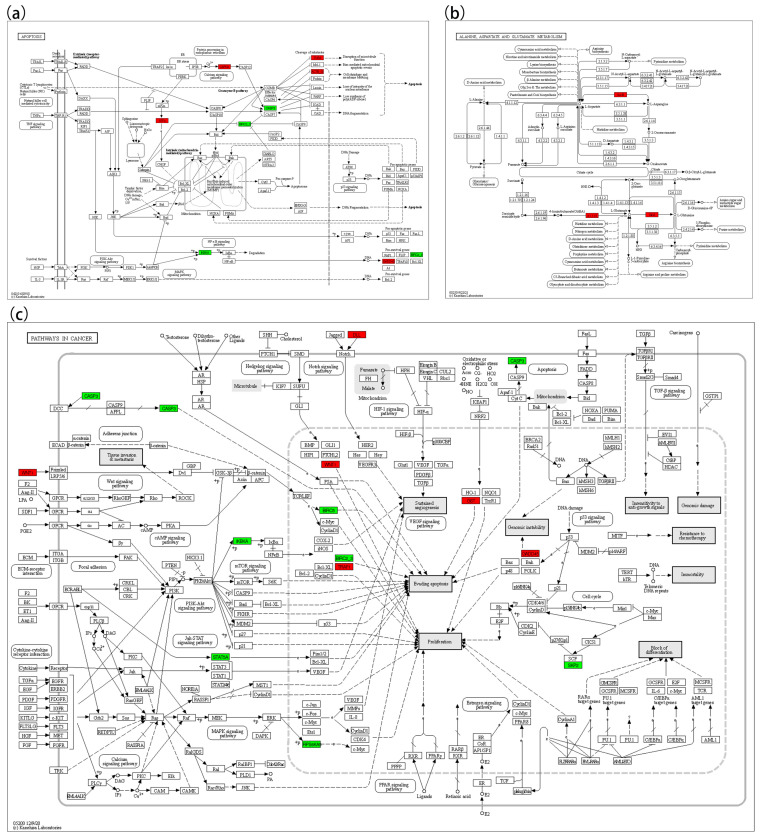
KEGG map 2. (**a**) Represents Apoptosis (map4010); (**b**) represents the Alanine, Aspartate and Glutamate Metabolism pathway (map00250); (**c**) represents Pathways in cancer (05200).

In the lipid oxidation pathway (Figure 6a), key upregulated genes included *LDLR*, *APOB*, *ABCA1*, *TLR2*, *MYD88*, *TRAF3*, *ATF4*, and *XBP1*; downregulated genes included *LBP*, *ABCG1*, *IKBKA*, and *CASP3.* In the TNF signaling pathway (Figure 6b), upregulated genes included *TRAF3* and *CREB1*; downregulated genes included *BIRC2/3*, *MAP3K8*, *IKBKA*, *CASP3*, *RPS6KA5*, and *IKBKG*. In the NF-κB pathway (Figure 6c), *MYD88*, *TRAF3*, and *GADD45* were upregulated, whereas *BIRC2/3*, *IKBKA*, and *LBP* were downregulated. In the apoptosis pathway (Figure 7a), *ATF4*, *CAPN1*, *TUBA*, *ACTB-G*, and *GADD45* were upregulated, while *IKBKA*, *BIRC2/3*, and *CASP3* were downregulated. In the cancer pathway (Figure 7c), five genes (*WNT1*, *DLL*, *TRAF1*, *GST*, and *GADD45*) were upregulated, while *CASP3*, *IKBKA*, *STAT5A*, *RPS6KA5*, *BIRC5*, *BIRC2/3*, and *SKP2* were downregulated.

### 3.6. qRT-PCR and RNA-Seq

**Figure 8:**
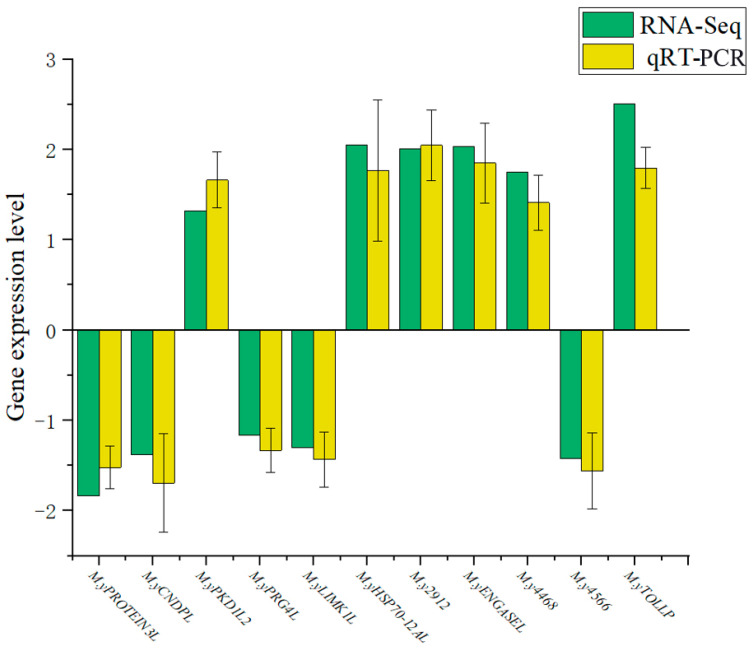
qRT-PCR validation of differentially expressed genes.

The differential expression of genes was validated by qRT-PCR. The gene validation results indicated that the relative expression levels of 11 tested genes measured by qRT-PCR were generally consistent with the trends observed in the RNA-Seq data (Figure 8).

## 4. Discussion

### 4.1. Antioxidant Response

In a sustained low-salinity environment, *Mizuhopecten yessoensis* is challenged not only by osmotic stress but also by complex, multilayered physiological responses. Due to being cultured in suspended cages, this species is particularly susceptible to salinity fluctuations, which can affect growth, energy metabolism, immune function, and gene expression. Salinity is a well-established ecological factor influencing bivalve adaptability; it regulates cellular osmosis and modulates multiple physiological pathways through signaling mechanisms, thereby influencing development, reproduction, and stress resistance.

After 60 days of low-salinity exposure, the antioxidant capacity of the scallops in the S26 group changed markedly compared to the S33 group. T-AOC and key antioxidant enzyme activities, such as those of superoxide dismutase (SOD) and peroxidase (POD), increased, while malondialdehyde (MDA) levels also rose, suggesting oxidative stress induced by low salinity [5]. Osmotic imbalance under low-salinity conditions can lead to excessive reactive oxygen species (ROS) production [26,27], which in turn activates antioxidant enzymes that scavenge superoxide radicals and hydrogen peroxide [28]. Transcriptomic data further showed upregulation of glutathione S-transferase (GST), an enzyme involved in ROS detoxification [29,30]. In addition, stress-related transcription factors ATF4 and XBP1 were upregulated, potentially indicating endoplasmic reticulum (ER) stress activation [30]. The unfolded protein response (UPR) initiated by ER stress can enhance molecular chaperone expression and elevate antioxidant defenses, acting synergistically with classical antioxidant enzymes [30].

Nevertheless, the elevated MDA levels indicate that ROS generation may have outpaced elimination, leading to oxidative damage [27]. When ROS production exceeds the antioxidant capacity, lipid peroxidation ensues, resulting in MDA accumulation and potentially initiating apoptosis [26,30]. These findings suggest that while the scallops activated antioxidant defenses in response to chronic low-salinity stress, a persistent redox imbalance still occurred. This pattern can be interpreted through the lens of the hormetic model, which offers a meaningful framework for understanding oxidative stress responses in bivalves under salinity fluctuations. According to this model, moderate increases in ROS can stimulate protective adaptations, including the upregulation of antioxidant enzymes—a trend reflected in the enhanced SOD, CAT, and POD activities observed under moderate salinity reduction. However, when salinity drops below a critical threshold, the balance shifts. The sharp elevation in MDA levels, together with signs of impaired enzyme function, suggests that ROS accumulation exceeded cellular tolerance, pushing the system from adaptation into damage. As Hong noted, ROS at physiological levels play crucial roles in signaling and homeostasis, but excessive ROS accumulation transforms their role from functional to cytotoxic [31].

Therefore, the redox state of scallops under chronic low-salinity stress appears to follow a biphasic response curve: initial activation of defenses under tolerable stress, followed by oxidative injury as stress intensity surpasses adaptive limits. This insight underscores the importance of identifying salinity thresholds beyond which protective mechanisms begin to fail.

### 4.2. Regulation of Apoptosis Signaling

Chronic low-salinity stress also affected apoptotic pathways. Transcriptomic data revealed significant changes in apoptosis-related gene expression in the S26 group. CASP3, a key executor of apoptosis, was downregulated, indicating suppression of the terminal apoptotic cascade, possibly to prevent excessive cell death [32]. Simultaneously, genes from the inhibitor of apoptosis protein (IAP) family, including *BIRC2*, *BIRC3*, and *BIRC5*, were also downregulated. Although IAPs normally inhibit caspase activity to prevent premature apoptosis [33], their downregulation may allow for the selective clearance of irreparably damaged cells, while the concurrent downregulation of *CASP3* raises the apoptotic threshold [34,35]. This dual-regulation strategy may help maintain tissue homeostasis under stress.

Additionally, *GADD45*, a stress-responsive gene, was upregulated. *GADD45* family members respond to osmotic and oxidative stress by inducing cell cycle arrest and DNA repair [36], and they modulate cell fate through interactions with kinases such as p38 and JNK [37]. *GADD45* may thus promote cell survival via repair or facilitate programmed cell death depending on the severity of damage.

Alterations were also observed in the NF-κB signaling pathway. The expressions of IKK complex components *IKBKA* and *IKBKG*, which are essential for NF-κB activation, were downregulated in the S26 group. NF-κB signaling typically promotes cell survival and inflammatory responses by inducing anti-apoptotic genes [38]. Its suppression, along with decreased *CASP3* and IAP expressions, suggests a finely tuned apoptotic control mechanism [39].

*CAPN1* upregulation, together with increased expressions of cytoskeletal proteins such as alpha-tubulin and *ACTB-G*, suggests cytoskeletal remodeling and involvement of calcium signaling. Calpain, a calcium-dependent protease, participates in protein turnover and stress signaling, potentially aiding in both apoptosis and structural maintenance [40]. The upregulation of cytoskeletal components may help stabilize cell structure under osmotic stress [30]. Collectively, these findings suggest that under chronic low-salinity conditions, *M. yessoensis* reprogrammed apoptotic signaling to balance cell survival and the removal of damaged cells [41].

### 4.3. Adaptation of Energy Metabolism

*M. yessoensis* also exhibited metabolic adaptations in response to prolonged low-salinity stress. Increased NAD-MDH activity in the S26 group indicates enhanced aerobic metabolism via the TCA cycle [42,43]. Energy is required to maintain osmotic homeostasis, including Na^+^/K^+^-ATPase activity and osmolyte synthesis. The upregulation of TCA cycle activity likely supported this elevated ATP demand [44,45,46]. Similar responses have been reported in other euryhaline bivalves, where low salinity stimulates fatty acid β-oxidation [44].

Transcriptome data also showed differential expression of lipid metabolism genes, particularly in the “lipid and atherosclerosis” pathway. Upregulation of *LDLR* and *APOB* suggests enhanced lipid uptake and redistribution [47], while increased *ABCA1* expression promotes cholesterol efflux. Interestingly, *ABCG1* was downregulated, suggesting a preferential reliance on *ABCA1*-mediated transport under stress. Such lipid transport adjustments may facilitate the removal of oxidized lipids, stabilize membranes, and deliver energy substrates to peripheral tissues.

Membrane remodeling likely contributed to osmoregulation. Exposure to low salinity can cause osmotic swelling or shrinkage, and shifts in lipid composition help preserve membrane fluidity [44]. Upregulation of genes involved in glycerophospholipid metabolism is consistent with previous reports in bivalves under salinity stress [47].

Energy allocation strategies also shifted. Downregulation of growth- and proliferation-related genes such as *STAT5A* and *SKP2* suggests reduced energy investment in growth and a reallocation toward survival and repair processes [48,49]. Meanwhile, *CREB1* was upregulated in the S26 group, potentially compensating for reduced NF-κB signaling and helping to maintain energy and survival-related gene expression [50,51].

Stress-responsive transcription factors *ATF4* and *XBP1*, upregulated under ER stress, may support not only antioxidant defense but also metabolic adjustments through the regulation of protein synthesis and lipid metabolism [52,53]. These changes collectively indicate a shift from a “growth mode” to a “maintenance mode” under chronic low-salinity stress [54].

Finally, enrichment of the “Pathways in cancer” category merits attention. Genes such as *WNT1* and *DLL*, typically associated with cell proliferation and differentiation, were upregulated. Wnt and Notch signaling may contribute to tissue repair or immune regulation under stress [55]. Although these pathways are also implicated in tumorigenesis, their expressions in adult scallops likely reflect controlled activation aimed at maintaining homeostasis. Similarly, *TRAF1* upregulation may serve as a compensatory mechanism for reduced IAP expression, ensuring survival signaling remains functional [56,57].

## 5. Conclusions

This study investigated the physiological and transcriptomic responses of *M. yessoensis* during 60 days of chronic low-salinity stress. The findings revealed phase-dependent adaptive mechanisms involving antioxidative regulation, apoptosis control, and metabolic remodeling. At moderate salinity reduction (28‰), enhanced activities of SOD, POD, and T-AOC, together with the upregulation of genes such as *GST*, *ATF4*, and *XBP1*, indicated the activation of antioxidant defenses and effective stress adaptation. In contrast, the 26‰ group showed reduced antioxidant enzyme activities and a high mortality rate (46%), suggesting that the adaptive mechanisms had reached their limits, leading to oxidative imbalance and physiological dysfunction.

Selective apoptosis regulation was observed through upregulation of *GADD45* and *CAPN1* and downregulation of *CASP3* and *BIRC2/3*, facilitating damaged cell clearance while minimizing excessive cell loss. Modulation of NF-κB signaling, with upregulation of *TRAF3* and downregulation of *IKBKA* and *IKBKG*, may contribute to the control of chronic inflammation. Metabolically, elevated NAD-MDH activity and upregulated expression of lipid metabolism genes (*ABCA1*, *LDLR*) and amino acid metabolism genes (*asnB*, *GlsA*, *GABA-T*) suggested enhanced aerobic respiration and nitrogen regulation under moderate stress.

In summary, *M. yessoensis* exhibited conditional physiological plasticity under chronic low-salinity stress, but this adaptability diminished as salinity dropped below a critical threshold, resulting in oxidative damage and increased mortality. These findings help define salinity conditions that pose physiological risks, providing practical guidance for improving scallop health and survival in variable aquaculture environments.

## Figures and Tables

**Table 1:** Real-time PCR primers used in the present study.

Gene Name	Primer Sequence	Gene ID
*M.yPROTEIN3L*	F: CATTGCGTACTCCCCACCTT R: GACCAATGAGCGACAGGACA	LOC110440822
*M.yCNDPL*	F: TCTTTCCGCATTCTTTTCCTCT R: ACTTTGGATTGAGAGAGATTGAAGG	LOC110440951
*M.yPKD1L2*	F: TCGCTTCCTATTCTGGGGGA R: ACAAAGTCCCAAAACACGGC	LOC110441234
*M.yPRG4L*	F: ACCCCTGAAAATGCAGCAGA R: TTCTCTTGACGGGTGAAGGC	LOC110441594
*M.yLIMK1L*	F: CAGGACAGGGGAGTGAAACA R: GCTCCTGTTCGTCACTCTCC	LOC110441862
*M.yHSP70-12AL*	F: GCTGGTTGGCAAATTCTCGG R: AGTGATGCATGTCCTACCGA	LOC110442164
*M.y2912*	F: ACCTGATACCGACAAGCATATT R: CAGGAAATGCTTTTTAACTTTGCGT	LOC110442912
*M.yENGASEL*	F: ACCTGATACCGACAAGCATATT R: CAGGAAATGCTTTTTAACTTTGCGT	LOC110443190
*M.y4468*	F: TGCTTGTACATACCAGGGTCA R: ACATGGGTTGATGGAGATGACC	LOC110444468
*M.y4566*	F: GCAACATTAAGACCATTTTGAGGATT R: TCATTCTGACAGTAAAAGTTGGCA	LOC110444566
*M.yTOLLP*	F: CTCCCCCGAGTTTCTCAAAG R: TCGAGACTTCCAAGGTTCCT	LOC110445648
*18S*	F: CTTCGAAGGCGATCAGATAC R: CTGTCAATCCTCACTGTGTC	LOC110442844

**Table 2:** Key genes in the key KEGG pathways.

Gene Name	KEGG Pathway ID	Trend
*CASP3*	5200, 5417, 04210, 04668	↓
*IKBKA*	5200, 5417, 0421, 04064, 04668	↓
*BIRC2-3*	04064, 5200, 04668, 04668	↓
*LBP*	04064, 5417	↓
*MYD88*	04064, 5417	↑
*TRAF3*	04064, 5417, 04668	↑
*ATF4*	04210, 5417	↑
*GADD45*	5200, 04064, 4210	↑
*glnAGLU*	00250	↑
*gadB*	00250	↑
*ASNS*	00250	↑

Note: The KEGG Pathway IDs correspond to the following pathway names: 04210 represents Apoptosis, 04668 represents the TNF signaling pathway, 05417 represents Lipid and atherosclerosis, 04064 represents the NF-kappa B signaling pathway, 00250 represents Alanine, aspartate and glutamate metabolism, and 05200 represents Pathways in cancer. These pathways are primarily involved in key biological processes such as apoptosis, inflammatory responses, immune signal transduction, and metabolic regulation.

## Data Availability

The original contributions presented in the study are included in the article; further inquiries can be directed to the corresponding authors.
